# Character of Cu and O in partially oxidized “29” Cu_*x*_O/Cu(111) from Auger Photoelectron Coincidence Spectroscopy

**DOI:** 10.1021/jacs.6c08886

**Published:** 2026-07-31

**Authors:** Swarnshikha Sinha, Danilo Kühn, Fredrik O. L. Johansson, Andreas Lindblad, Pavel A. Korzhavyi, Nils Mårtensson, Alexander Föhlisch

**Affiliations:** † Institute for Methods and Instrumentation for Synchrotron Radiation Research, 28340 Helmholtz-Zentrum Berlin für Materialien und Energie GmbH, Albert-Einstein Straße 15, Berlin 12489, Germany; ‡ Institut für Physik und Astronomie, Universtät Potsdam, Karl-Liebknecht- Straße, Potsdam 14476, Germany; § Uppsala-Berlin Joint Laboratory on Next Generation Photoelectron Spectroscopy, Berlin 12489, Germany; ∥ Division of X-ray Photon Science, Department of Physics and Astronomy, Uppsala University, P.O. Box 256, Uppsala 751 20, Sweden; ⊥ Department of Materials Science and Engineering, KTH Royal Institute of Technology, Brinellvägen 23, Stockholm 100 44, Sweden

## Abstract

The controversial formal oxidation state of partially oxidized copper has been determined by the enhanced surface and chemical selectivity of Cu-L_3_VV, Cu-M_3_VV, and O–KVV Auger photoelectron coincidence spectroscopy (APECS) and first-principles calculations. We compare metallic Cu(111) of formal oxidation state (0), Cu_2_O cuprous Cu­(I) oxide, and CuO cupric Cu­(II) oxide to partially oxidized “29” Cu_*x*_O/Cu­(111). The oxidation state of Cu within the top three layers of “29” Cu_*x*_O/Cu­(111) is found as Cu(0.3) close to the formal Cu(0) oxidation state of metallic Cu(111). A distribution of oxygen chemical species is observed in agreement with the proposed three types of oxygen bonding. This clarifies essential steps for copper-based materials and chemistry by the enhanced selectivity of APECS made applicable to materials.

## Graphical abstract

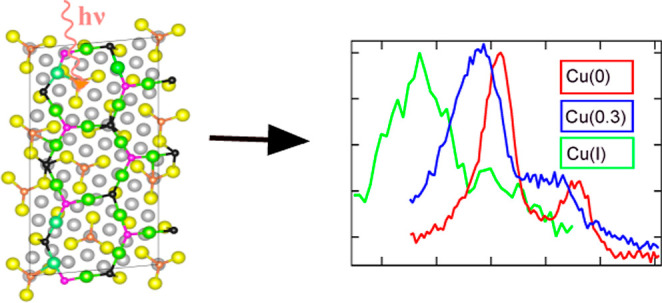

## 1 Introduction

Partially oxidized Cu surfaces are formed upon the exposure of metallic Copper Cu(0) to oxygen, being a precursor of bulk Cu_2_O cuprous Cu­(I)­oxide and CuO cupric Cu­(II)­oxide. They play an essential role in the formation of protective layers hindering corrosion for technical applications, electronic devices, or to withstand long-term chemical modifications in nuclear waste storage containment environments.^2,17–19,34,36,39,43,49^ Partially oxidized Cu surfaces also contribute significantly in heterogeneous catalytic processes over copper catalysts.^1,6,11^ On single-crystal Cu(111) surfaces, fully and partially oxidized layers form by exposure to gaseous oxygen. The fully oxidized surface of a Cu_2_O thin film on Cu(111) consists of fused hexagonal rings with a periodicity of 0.6 nm and occupying a surface area of 0.316 nm^2^. Each ring contains buckled, linear O–Cu–O bonds with elevated and lowered oxygen atoms.^13,16,35,46^ The formation of partially oxidized Cu(111) surfaces is highly sensitive to O_2_ pressure and Cu substrate temperature.^5,13,23,28,29,31,33,38,40,44,46,47^ A characteristic feature is the formation of large unit cells. On Cu(111), partially oxidized surface reconstructions are found with a 29 times larger unit cell than the clean Cu(111) surface, denoted as “29” Cu_*x*_O/Cu­(111) and even with a 44 times larger unit cell, denoted as “44” Cu_*x*_O/Cu­(111). These “44” and “29” structures have been proposed to involve eight and six hexagonal rings per oxide unit cell, respectively.^13,23,28^ The “29” top layer resembles locally a 16% compressed, highly strained Cu_2_O­(111) surface unit with an Oxygen-to-Cu concentration larger than Cu_2_O­(111).^10,46^ Out of the six hexagonal rings of the “29” unit cell, sometimes five or all six have been proposed to host a 3-fold coordinated oxygen adatom. The buckled hexagonal rings have elevated 3-fold coordinated surface oxygen (O) atoms and lowered 4-fold coordinated subsurface oxygen atoms.^23,46^ A Woods notation of $\left(\sqrt{13}R46.1^{{}^{\circ}}\times 7R21.8^{{}^{\circ}}\right)$ Cu_*x*_O/Cu­(111) has been proposed with a lattice match of 99% to Cu(111).^45^ This strained “29” Cu_*x*_O/Cu­(111) surface oxide relaxes with increasing oxygen dosage by oxygen diffusion into the bulk resulting in Cu_2_O/Cu­(111) thin film formation.^12^

From an electronic structure perspective, it has been challenging to identify differences in the local Cu valence state of clean Cu(111) and the partially oxidized “29” Cu_*x*_O/Cu­(111) by core-level photoelectron or Auger electron spectroscopy as reported previously.^25^ This study suggests the formal oxidation state of partially oxidized surface oxide remains closer to Cu(0) of metallic copper. These findings are highly puzzling since the number of oxygen atoms per Cu atom within the “29” Cu_*x*_O/Cu­(111) top layer exceeds stoichiometric Cu_2_O cuprous Cu­(I)­oxide toward CuO cupric Cu­(II)­oxide, which should indicate a formal oxidation state above (I).^10,26,46,48^ Auger photoelectron coincidence spectroscopy (APECS)^24^ allows an unrivaled surface sensitivity^22^ and chemical state differentiation^15^ over X-ray absorption, photoelectron, or Auger electron spectroscopy.

In this work, we determine the oxidation state character of Cu and the chemical state distribution of oxygen within the partially oxidized “29” Cu_*x*_O/Cu­(111) surface oxide in direct comparison to Cu(111) and in situ grown bulk-like films of Cu_2_O and CuO. We examine the local valence configurations of the “29” Cu_*x*_O/Cu­(111) surface oxide with Cu 2p_3/2_-L_3_VV, Cu 3p-MVV, and O 1s-KVV Auger photoelectron coincidence spectroscopy. We find that the “29” Cu_*x*_O/Cu­(111) surface oxide has an oxidation state of Cu(0.3), which is closer to metallic Cu(0) than Cu­(I) cuprous oxide. We also model the spectral finds with two distinct oxygen adatom configurations derived from first-principles theory: the 6 O adatom model and the 5 O adatom model. In the 6 O adatom model, all 6 Cu–O rings within the surface unit cell are occupied by O adatoms, and in the 5 O adatom model, only 5 of the 6 Cu–O rings are occupied by O adatoms. The Cu 3p-MVV two-hole spectra for both 6 and 5 O adatom models are analyzed with a depth-dependent intensity distribution, the (Z + 2) surface core level shifts (SCLS) from density functional theory (DFT) calculations, and Voigt line shapes. Within both 6 and 5 O adatom models, the first layer is copper bonded with two oxygen species, the second layer is copper bonded with a single oxygen species, with some adatom oxygen species, and third is the Cu(111) bulk of the “29” Cu_*x*_O/Cu­(111) surface oxide clustered around the average oxidation state of (0.3). We also observe a gradual chemical state distribution of the formally three different oxygen species within the “29” Cu_*x*_O/Cu­(111) surface oxide for both the 6 and 5 O adatom models.

## 2 Results and Discussion

### 2.1 Cu valence and oxidation state from Auger photoelectron coincidence spectroscopy in “29” Cu _*x*_O/Cu­(111) surface oxide vs Cu(111), Cu_2_O, and CuO

In Figure 1, Auger photoelectron coincidence (APECS) measurements performed at the Cu 2p_3/2_-L_3_VV edge of partially oxidized “29” Cu_*x*_O/Cu­(111) surface oxide are presented in direct comparison to Cu(111), bulk-like films of Cu_2_O and CuO, prepared by dissociative adsorption of O_2_ at various temperatures and pressures as described in detail in the experimental section. The as obtained two-dimensional (2D) true coincidence maps of the Cu 2p_3/2–_ L_3_VV edge are presented in *E*_kin_^AES^ + *E*_kin_^PES^ sum energy scale preserving the energy sum as the photoelectron, and Auger electron emission remains a coherent process (see the supporting Information and ref 42). In Figure 1a–d, 2p_3/2_/L_3_VV energy distribution maps of (Cu_2_O) cuprous oxide, “29” Cu_*x*_O/Cu­(111) surface oxide, and Cu(111) are similar but differ significantly for the (CuO) cupric oxide. CuO and Cu_2_O (panels (a), (b)) have been acquired with 1250 eV photon energy, whereas “29” Cu_*x*_O/Cu­(111) and Cu(111) (panels (c), (d)) with 1100 eV photon energy. This choice was made to include also the shakeup regions of Cu_2_O and CuO. Here, we focus solely on the APECS main line region (shown in panels (a–d)). From these, the two-hole final state (panel (e)) binding energies are derived, which are independent of photon energy (details in the Supporting Information). The differences in kinetic energies (a–d) are, however, reflecting the photon energies used.

**Figure 1:**
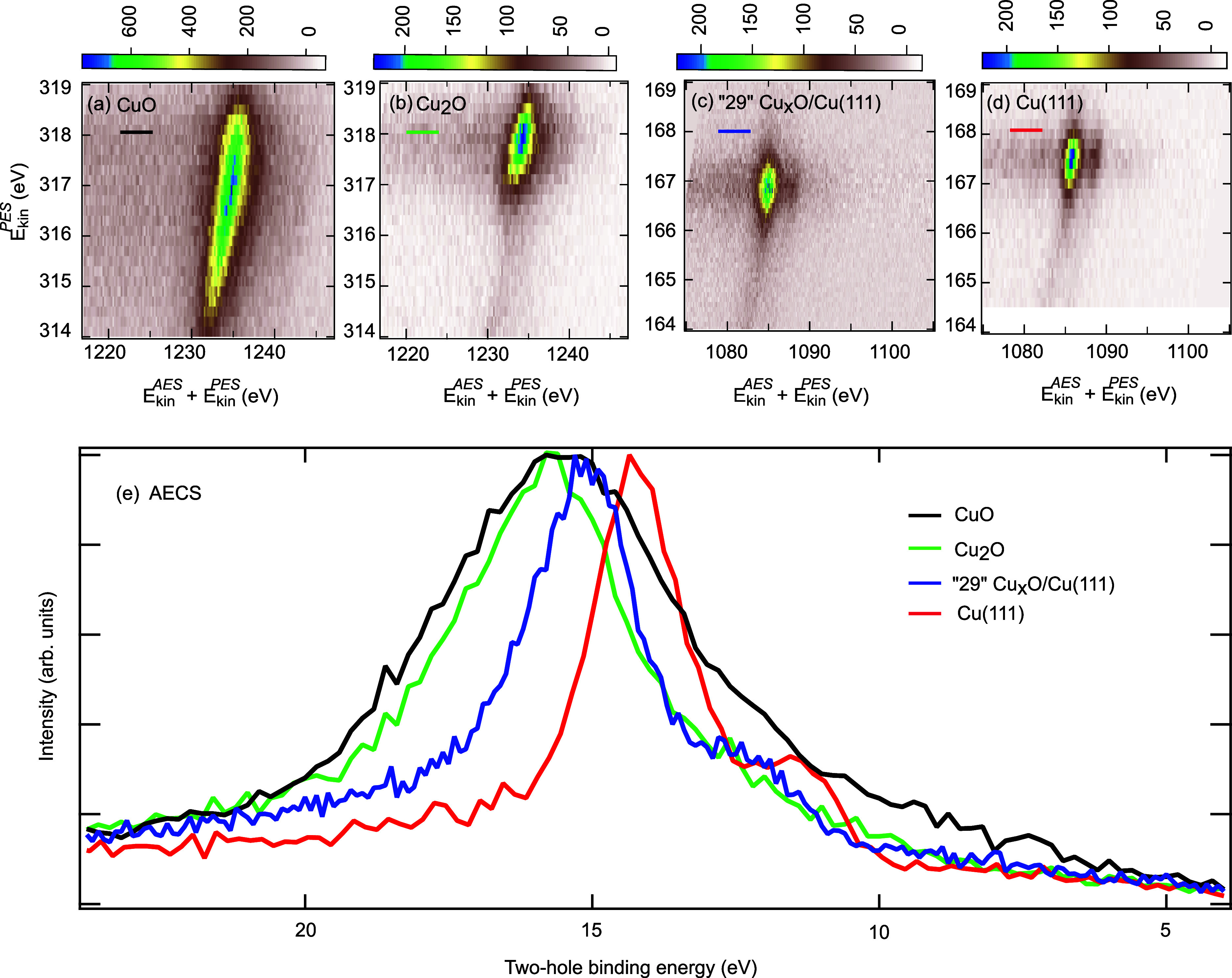
Cu 2p_3/2–_L_3_VV Auger photoelectron coincidence spectroscopy (APECS) maps of cupric oxide (CuO), cuprous oxide (Cu_2_O), “29” Cu_*x*_O/Cu­(111) surface oxide, and Cu(111) are presented in panels (a), (b), (c), and (d). Panel (e) shows one-dimensional Auger electron coincidence spectra (AECS) of cupric oxide (CuO), cuprous oxide (Cu_2_O), “29” Cu_*x*_O/Cu­(111) surface oxide, and Cu(111) on a two-hole binding energy scale obtained by integrating along the *E*_kin_^PES^ axis.

Within each map, the two-dimensional APECS data are integrated along the *E*_kin_^PES^ direction for the CuO (cupric oxide), Cu_2_O (cuprous oxide), “29” Cu_*x*_O/Cu­(111) surface oxide, and Cu(111), to obtain one-dimensional (1D) Auger Electron Coincidence Spectra (AECS) in Figure 1e. In particular, APECS allows discrimination against overlapping features from cascade processes, e.g., L_3_V–VVV following Coster–Kronig decay of Cu 2p_1/2_,^8^ and other unrelated electron background, which enables to extract the pure L_3_VV two-hole states.

The spectra in Figure 1e show a systematic binding energy shift between the Cu(111) surface, the “29” Cu_*x*_O/Cu­(111) surface oxide, and the two bulk oxides. As the formal oxidation states and the valence electronic structure of Cu(111) with oxidation state (0), Cu_2_O with oxidation state (I), and CuO with oxidation state (II) are known, we can relate and benchmark the “29” Cu_*x*_O/Cu­(111) surface oxide under investigation to these.

In Table 1, we give for our reference materials Cu(111), Cu_2_O, and CuO the respective valence electronic structure, oxidation state, and the spectroscopic Cu 3d two hole final states, screened by the itinerant and covalently interacting 4s and 4p electrons, which we denote as [4s^2−δ^4p^γ^] with fractional fillings 2 – δ and γ. Our APECS spectra show that the “29” Cu_*x*_O/Cu­(111) surface oxide is in binding energy between the oxidation state (0) Cu(111) and the oxidation state (I) Cu_2_O. As seen in Table 1, the spectroscopic final state of Cu(111) and Cu_2_O has near equivalent screened 3d^8^[4s^2−δ^4p^γ^] final states, whereas the oxidation state (II) CuO reaches a screened ${3d}^{8}\mathrm{L̅}$ final state, with L as the ligand hole of O 2p. This is because in the core-excited state of CuO, the main 2p_3/2_ line is fully screened by a O 2p-3d charge transfer leading to a final state differentiated in binding energy and shape.

**Table 1:** Electronic Structure Properties of Cu(111), Cu_2_O, and CuO Systems Investigated by Auger Photoelectron Coincidence Spectroscopy^a^

	Cu-oxidation state	Cu ground state valence	APECS two-hole final state
Cu(111)	0	3d^10^4s^1^	3d^8^[4s^2−δ^4p^γ^]
Cu_2_O	I	3d^10^4s^0^	3d^8^[4s^2−δ^4p^γ^]
CuO	II	3d^9^4s^0^	${3d}^{8}\mathrm{L̅}$

a Screening of the two-hole final state by itinerant and covalently interacting 4s and 4p electrons in the Cu(111) and Cu_2_O systems is denoted as [4s^2−δ^4p^γ^] with fractional fillings 2 – δ and γ. For CuO, the two-hole final state is denoted by ${3d}^{8}\mathrm{L̅}$ with L as the ligand hole of O 2p.

We thus see directly from binding energy and spectral shape that the “29” Cu_*x*_O/Cu­(111) surface oxide must also have a 3d^8^[4s^2−δ^4p^γ^] final state with a formal Cu-oxidation state between (0) and (I). From this observation, we can exclude Cu-oxidation states between (I) and (II) for the “29” Cu_*x*_O/Cu­(111) surface oxide. To quantify the oxidation state of “29” Cu_*x*_O/Cu­(111) surface oxide, we zoom into the detailed shifts between Cu(111), the “29” Cu_*x*_O/Cu­(111) surface oxide, and Cu_2_O reaching identical 3d^8^[4s^2−δ^4p^γ^] final states by measuring Cu 3p-MVV APECS. Here, the kinetic energy of the auger and photoelectrons is close to the minimum of the universal curve of inelastic mean free path (IMFP). Hence, Cu 3p-MVV APECS has a smaller probing depth compared to Cu 2p-LVV APECS. For pure Cu(111), ≈69–70% of the signal originates from the topmost layer in the 3p-MVV measurement, whereas the 2p-LVV measurement provides only ≈60–62% from the top layer. The detailed comparison of the depth-dependent signal contributions for Cu 2p-LVV and Cu 3p-MVV APECS is discussed in ref 42

In Figure 2a–c, the APECS maps measured at the Cu 3p-MVV edge of Cu_2_O, the “29” Cu_*x*_O/Cu­(111) surface oxide, and Cu(111) are presented. Figure 2d shows AECS obtained by integrating the coincidence maps in 2­(a–c) along the *E*_kin_^PES^ direction, indicated by colored blocks in the maps. We exclude the Cu 3p_1/2_/MVV region from the integration to avoid inelastic loss contributions arising from inelastically scattered 3p_1/2_ electrons and Coster–Kronig decay.^42^ In Figure 2d, we observe for the “29” Cu_*x*_O/Cu­(111) surface oxide, spectral features are shifted toward higher binding energy, compared to the Cu(111) surface, but to lower binding energy than to Cu_2_O cuprous oxide. The line shape of “29” Cu_*x*_O/Cu­(111) herein also shows resemblance for a more metallic-like shape of Cu rather than to Cu_2_O cuprous oxide. In particular, the characteristic ^1^G (at about 14 eV) and ^3^F (at about 11 eV) peaks of the 3d^8^[4s^2−δ^4p^γ^] atomic multiplet of metallic copper are, though broadened, also clearly visible in the “29” Cu_*x*_O/Cu­(111) surface oxide, whereas Cu_2_O shows a smooth tailing of the atomic final state (at about 16.6 eV) toward lower two-hole binding energy (band-like region). This implies that the electronic structure of the copper atoms in the surface layer of the “29” Cu_*x*_O/Cu­(111) surface oxide resembles a set of chemically inequivalent copper species interacting with oxygen, however, with a local electronic structure close to metallic copper. Since Cu 3p_3/2_-M_3_VV APECS reaches near equivalent 3d^8^[4s^2−δ^4p^γ^] final states for Cu(111), the “29” Cu_*x*_O/Cu­(111) surface oxide, and Cu_2_O, the variation of binding energy reflects on directly to the ground state valence electronic structure and formal Cu-oxidation state present in these three systems. The fractional filling parameters δ and γ here are labeled notations for 4s and 4p occupancy, and the two-hole final state assignments are physically motivated by the different screening mechanisms of the Cu(0), Cu(0.3), Cu­(I), and the strongly correlated Cu­(II) system. We can therefore relate the observed chemical shifts to valence occupation and oxidation state linking binding energy to occupation number.

**Figure 2:**
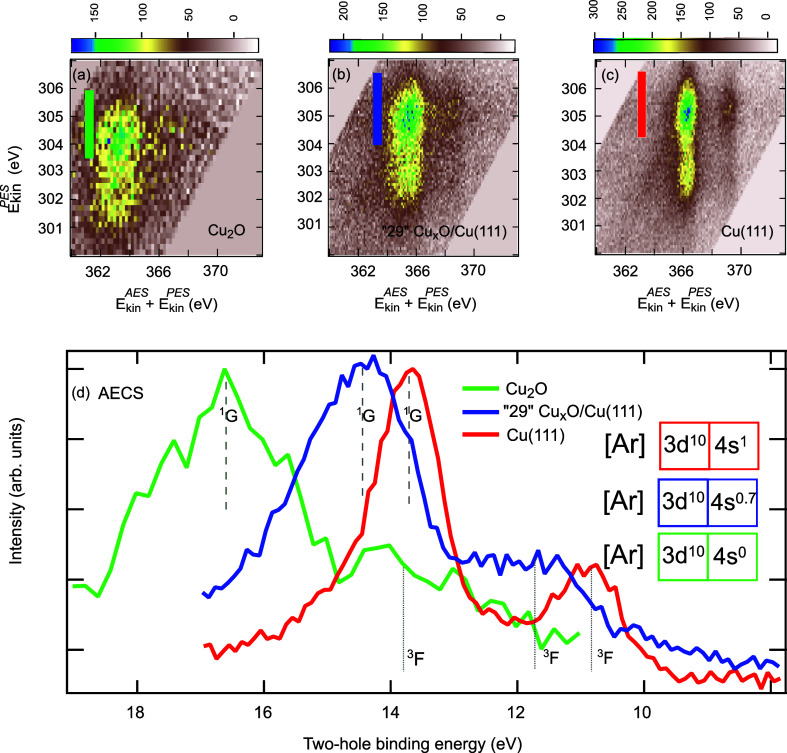
Cu 3p-MVV Auger photoelectron coincidence spectroscopy (APECS) maps for cuprous oxide (Cu_2_O), partially oxidized “29” Cu_*x*_O/Cu­(111) surface oxide, and Cu(111), in panels (a), (b), and (c). Panel (d) features Cu 3p_3/2–_ M_3_VV 1-dimensional Auger electron coincidence spectra (AECS) on the two-hole binding energy scale for the cuprous oxide (Cu_2_O), “29” Cu_*x*_O/Cu­(111), and Cu(111) obtained by integrating the APECS maps along the *E*_kin_^PES^ direction depicted by green, blue, and red blocks. Cu ground state valence configuration for the cuprous oxide (Cu_2_O), partially oxidized “29” Cu_*x*_O/Cu­(111) surface oxide, and Cu(111) are also presented. Electronic structure information is derived (see main text) with variation in screening due to the interaction in covalent and itinerant 4s and 4p orbitals of the Cu(111), “29” Cu_*x*_O/Cu­(111) surface oxide, and cuprous oxide (Cu_2_O).

We determine from the experimental Cu 3p_3/2–_ M_3_VV AECS spectra in Figure 2 (d) the ^1^G experimental multiplet peak positions of the 3d^8^[4s^2−δ^4p^γ^] two hole final states by a Gaussian fit and locate peak maxima of Cu(111) at 13.70 ± 0.01 eV, Cu_2_O at 16.59 ± 0.05 eV, and “29” Cu_*x*_O/Cu­(111) at 14.38 ± 0.05 eV. Since metallic Cu(111) has with its 3d^10^4s^1^ occupancy the formal oxidation state (0) and Cu_2_O with 3d^10^4s^0^ occupancy oxidation state (I), we can therefore assign state occupancy and oxidation state for the “29” Cu_*x*_O/Cu­(111) surface oxide in linear interpolation. Thus, “29” Cu_*x*_O/Cu­(111) surface oxide has an occupancy of 3d^10^4s^0.7^ and a formal Cu-oxidation state of (0.3). Table 2 summarizes the results of valence and formal Cu-oxidation state for Cu(111), the “29” Cu_*x*_O/Cu­(111) surface oxide, and Cu_2_O. This oxidation state reflects the partial charge transfer from copper to oxygen in the partially oxidized “29” Cu_*x*_O/Cu­(111) surface oxide. It is also important to note that the spectral features in the AECS spectra of the “29” Cu_*x*_O/Cu­(111) surface oxide are gradually broadened in comparison to Cu(111). This indicates that the complex “29” Cu_*x*_O/Cu­(111) surface oxide has different but near-equivalent Cu sites clustered around the formal Cu-oxidation state of (0.3).

**Table 2:** Electronic Structure Properties of “29” Cu_*x*_O/Cu­(111) Derived from Auger Photoelectron Coincidence Spectroscopy with Respect to Cu(111) and Cu_2_O Systems^a^

	Cu-oxidation state	Cu ground state valence	APECS two-hole final state
Cu(111)	0	3d^10^4s^1^	3d^8^[4s^2−δ^4p^γ^]
“29” Cu_*x*_O/Cu(111)	0.3	3d^10^4s^0.7^	3d^8^[4s^2−δ^4p^γ^]
Cu_2_O	I	3d^10^4s^0^	3d^8^[4s^2−δ^4p^γ^]

a Screening of the two-hole final state by itinerant and covalently interacting 4s and 4p electrons is denoted as [4s^2−δ^4p^γ^] with fractional fillings 2 – δ and γ.

### 2.2 Computation model of the “29” Cu_*x*_O/Cu­(111) surface oxide and its Cu 3p_3/2_-M_3_VV 3d^8^[4s^2‑δ^4p^γ^] ^1^G, ^3^F Auger photoelectron coincidence spectral features

To advance our understanding of Cu and Oxygen sites for the “29” Cu_*x*_O/Cu­(111) surface oxide, we conducted first-principles calculations with density functional theory (detailed information provided in the Experimental section). We model two different surface structures to investigate the robustness of our approach: First is a situation where all the 6 Cu–O rings of the unit cell are being occupied with O adatoms, denoting it as the 6 O adatom model. Then is the 5 O adatom model situation where only 5 of the 6 Cu–O rings being occupied by O adatoms by the removal of 1 O adatom (and placing it to the gas phase as a 0.5*O2 molecule). It is noteworthy that this does not lower the total energy, indicating that the full adatom layer is energetically more stable (about 1 eV per O atom). However, entropy of the gas phase may change the free energy balance: at room temperature, the structure with 5 adatoms should then be in equilibrium with very low O partial pressure. The structure-optimized models are represented in Figure 3. This study indicates the presence of three Cu entities categorized as three layers. The copper atoms of the first layer (Cu 1, green) are chemically bonded to oxygen atoms from top and bottom. The second layer copper atoms (Cu 2, yellow) are bonded only from top to the oxygen atoms with some oxygen adatoms additionally present. The third copper layer (Cu 3, gray) is not coordinated to oxygen and hence similar to bulk copper atoms. For these models of the commensurate “29” Cu_*x*_O/Cu­(111) surface oxide, we obtained chemical shifts of the various Cu species in the different Cu layers relative to bulk Cu(111). We call these as surface core level shifts (SCLS). The screened 3d^8^[4s^2−δ^4p^γ^] final states are treated within the equivalent (Z + 2) impurity approximation as in our previous work on Cu(111), Cu(110), and Cu(100).^42^ Within this model, the (Z + 2) SCLS chemical shifts of the various Cu species are established for the 6 O adatom model as Cu 1 at (+0.171) ± 0.295 eV, Cu 2 at (−0.188) ± 0.138 eV, and Cu 3 at (−0.005) ± 0.013 eV and for the 5 O adatom model as Cu 1 at (+0.147) ± 0.352 eV, Cu 2 at (−0.124) ± 0.107 eV and Cu 3 at (+0.011) eV. In both cases, the SCLS value obtained for Cu 3 is very small, and it shows that Cu 3 is already bulk like. We thus differentiate from here on Cu 1 and Cu 2 and treat Cu­(3 + Bulk) together. Further, we derive relative atomic density of Cu 1, Cu 2, and Cu (3 + Bulk) atoms to develop a depth-dependent intensity model for the “29” Cu_*x*_O/Cu­(111) surface oxide. The depth-dependent intensity contributions are based on the calculated mean escape depths (MED) of the coincidence electron pairs for each layer of Cu species and the relative atomic density in “29” Cu_*x*_O/Cu­(111) surface oxide (see the Supporting Information^41^ and our previous work^22^). These are then combined with Voigt line shapes and (Z + 2) SCLS chemical shifts to model the “29” Cu_*x*_O/Cu­(111) surface oxide experimental data. With this approach, we model the Cu 3p_3/2–_M_3_VV spectra of “29” Cu_*x*_O/Cu­(111) surface oxide (blue) as the ^1^G and ^3^F multiplet of the screened two-hole final state Cu 3d^8^[4s^2−δ^4p^γ^] and as a weighted superposition of Cu 1, Cu 2, and Cu (3 + Bulk). The fit results of this model are presented with the experimental data in Figure 3 for the 6 and 5 O adatom models. The ^1^G and ^3^F components of the first layer Cu 1 are shown as green solid lines, the second layer Cu 2 as yellow solid lines, and Cu (3 + Bulk) as gray solid lines. Experimental and lifetime broadening are given by Gaussian and Lorentzian contributions. The Lorentzian lifetime broadening for the ^1^G and ^3^F peaks for all three layers is fixed at 0.76 eV (fwhm), motivated from our previous study of Cu surfaces.^42^ Gaussian (fwhm) is found to be 1.5 ± 0.03 eV (fwhm) for the 6 O adatom model and 1.47 ± 0.03 eV (fwhm) for the 5 O adatom model. This higher than experimental broadening of 0.3 eV in both the 6 and 5 O adatom models might be associated with, i.e., vibrational effects and variation of the local potential. Also the wide standard deviation distribution of the (Z + 2) SCLS for the 6 and 5 O adatom model can contribute to this broadening. The energy splitting of the ^1^G and ^3^F components is an atomic property of Cu 3d^8^ and thus identical for the three layers in the 6 and 5 O adatom model and has a value of 2.80 ± 0.01 eV. The multiplet intensity ratio of ^3^F/^1^G is at 0.32 ± 0.01 in good agreement with our previous study of metallic Cu(111).^42^ The position and the two-hole binding energies are given in Table 3 for the 6 O adatom model: The Cu 3d^8^[4s^2−δ^4p^γ^] ^1^G and ^3^F peak positions for the three layers are 14.53 eV for the ^1^G component of Cu 1 and 11.73 eV for ^3^F. For Cu 2, it is 14.16 eV for the ^1^G component and 11.36 eV for the ^3^F component. The ^1^G and ^3^F component peak positions for the Cu (3 + Bulk) component are 14.35 and 11.55 eV, respectively. The position and the two-hole binding energies are given in Table 4 for the 5 O adatom model: The Cu 3d^8^[4s^2−δ^4p^γ^] ^1^G and ^3^F peak positions, for the three layers, are 14.49 eV for the ^1^G component of Cu 1 and 11.69 eV for ^3^F. For Cu 2, it is 14.22 eV for the ^1^G component and 11.42 eV for the ^3^F component. The ^1^G and ^3^F component peak positions for the Cu (3 + Bulk) component are 14.35 and 11.55 eV, respectively. The highly precise match between the experiment and model as seen in Figure 3 (b) for both the 6 and 5 O adatom model shows that the “29” Cu_*x*_O/Cu­(111) surface oxide is a superposition of the three different copper site Cu 1, Cu 2, and Cu (3 + Bulk), all being close to metallic Cu and being clustered around the average oxidation state of (0.3).

**Figure 3:**
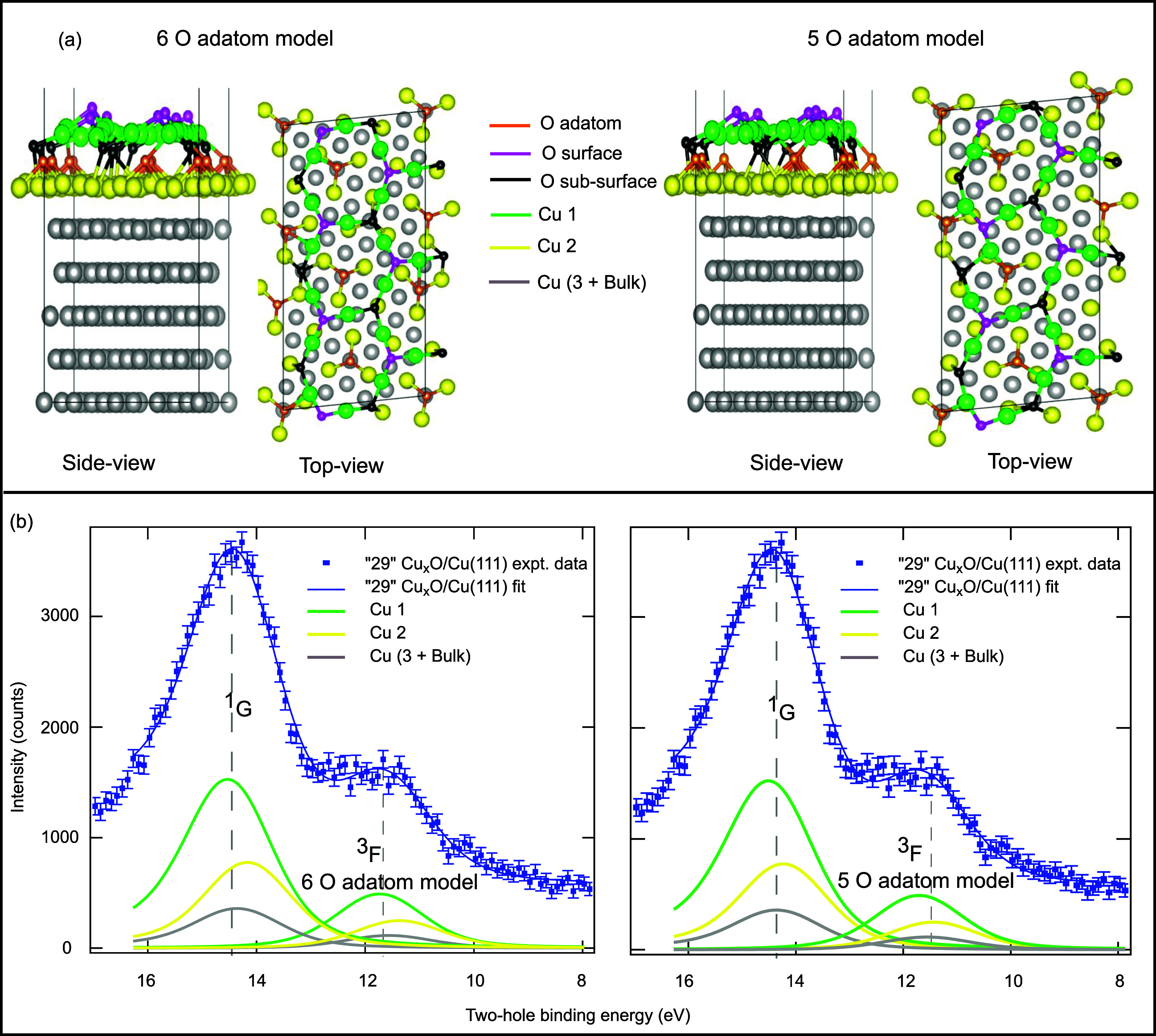
Computation model of the “29” Cu_*x*_O/Cu­(111) surface oxide and the resulting fit of the Cu 3p_3/2–_M_3_VV 3d^8^[4s^2−δ^4p^γ^] ^1^G and ^3^F multiplet in comparison to the experiment. To investigate the surface chemistry of both 6 O adatom model and 5 O adatom model generated after the removal of one O adatom out of the 6 Cu–O rings into the gas phase has been modeled. (a) Parameters and sites of the slab calculation. Green represents the Cu atoms of the first oxidic layer, yellow represents the Cu atoms of the second oxidic layer, and gray represents the Cu atoms of Layer 3 being actually highly equivalent to the underlying bulk. Pink and orange atoms are the surface and adatom oxygen species which are predominantly 3-fold coordinated for the 6 and 5 O adatom model. Black atoms are the subsurface oxygen which are 4-fold coordinated for both the 6 and 5 O adatom model. (b) Spectral fit to the experimental data of the “29” Cu_*x*_O/Cu­(111) surface oxide using the Z + 2 approximation computed two-hole binding energies of the ^1^G and ^3^F multiplet of Cu 3d^8^ screened by the itinerant and covalently interacting [4s^2−δ^4p^γ^] for Cu layers Cu 1, Cu 2, and Cu­(3 + Bulk). Line shape is given by a Voigt profile. Parameters in Table 3 for the 6 O adatom system and in Table 4 for the 5 O adatom system.

**Table 3:** Results of the “29” Cu_*x*_O/Cu­(111) Surface Oxide Fit of Cu 3p_3/2–_M_3_VV 3d^8^[4s^2−δ^4p^γ^] for the 6 O Adatom Model^a^

“29” Cu_*x*_O/Cu(111) surface oxide	fit values	“29” Cu_*x*_O/Cu(111) surface oxide	fit values	
			^1^G	^3^F
Gaussian	1.47	Cu 1	14.53	11.73
fwhm (eV)				
Lorentzian	0.76	Cu 2	14.16	11.36
fwhm (eV)				
Shirley	0.07	Cu (3 + Bulk)	14.35	11.55

a ^1^G and ^3^F multiplet shown in Figure 3.

**Table 4:** Results of the “29” Cu_*x*_O/Cu­(111) Surface Oxide Fit of Cu 3p_3/2_-M_3_VV 3d^8^[4s^2−δ^4p^γ^] for the 5 O Adatom Model^a^

“29” Cu_*x*_O/Cu(111) surface oxide	fit values	“29” Cu_*x*_O/Cu(111) surface oxide	fit values	
			^1^G	^3^F
Gaussian	1.50	Cu 1	14.49	11.69
fwhm (eV)				
Lorentzian	0.76	Cu 2	14.22	11.42
fwhm (eV)				
Shirley	0.07	Cu (3 + Bulk)	14.35	11.55

a ^1^G and ^3^F multiplet shown in Figure 3.

### 2.3 Oxygen species within the “29” Cu_*x*_O/Cu­(111) surface oxide: O 1s-KVV Auger photoelectron coincidences and the computational framework

To characterize the “29” Cu_*x*_O/Cu­(111) surface oxide from the perspective of the oxygen atoms, we performed a O 1s-KVV APECS investigation. Nominally, as discussed in the introduction section, the structural models of “29” Cu_*x*_O/Cu­(111) surface oxide indicate that the various oxygen species can be structurally clustered around three different bonding situations, adatom, surface, and subsurface oxygen.^9^ Figure 4a displays the experimental O 1s-KVV APECS data with a gradual variation of Auger and photoelectron energies indicating a variety of oxygen bonding. However, even with the enhanced surface and chemical selectivity of O 1s-KVV APECS, no distinct, separate spectral features are readily detectable in the 2D coincidence map. To quantify the gradually varying O 1s-KVV distribution further, we define three regions across the APECS spectral distribution highlighted in orange for the adatom oxygen, pink for the surface oxygen, and black for the subsurface oxygen following the assignment of a previous photoelectron study.^9^ We then integrate within each of them along the *E*_kin_^PES^ direction and yield Auger electron coincidence spectra (AECS) on the two-hole binding energy scale shown in Figure 4b. For each chemically shifted species, we find the ^1^D and component of the O 2p^4^ atomic multiplet with the following ^1^D peak positions: adatom oxygen (orange) at 16.5 eV, the surface oxygen (pink) at 16.7 eV, and the subsurface oxygen (black) at 16.9 eV. This shows the varying bonding strength of these species clearly. Next to the atomic multiplet, we observe between 6 and 10 eV the spectral distribution of itinerant band-like two-hole final states. Toward the Fermi level, we also provide the noncoincidence Auger electron spectrum (AES) as the spectral envelope of all three species (gray circles).

**Figure 4:**
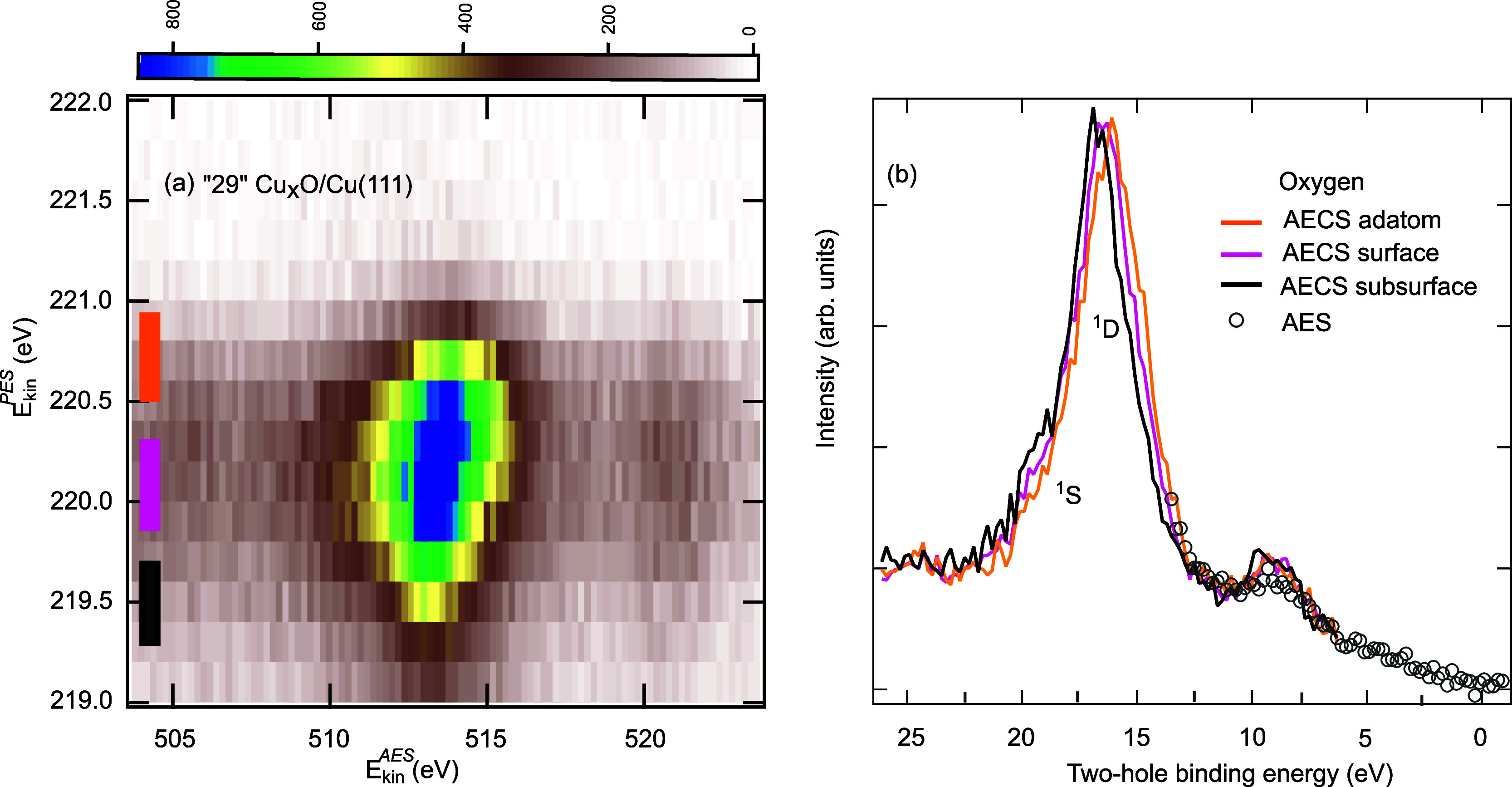
O 1s-KVV Auger photoelectron coincidence spectroscopy (APECS) maps of “29” Cu_*x*_O/Cu­(111) surface oxide and Auger electron coincidence spectral (AECS) projection for adatom, surface, and subsurface oxygen species. (a) O 1s-KVV APECS map with a gradual variation of Auger and photoelectron energies. Oxygen species assignment following^9^ adatom oxygen (orange), surface oxygen (pink), and subsurface oxygen (black). (b) O 1s-KVV Auger electron coincidence spectra (AECS) of the three oxygen species by integrating the adatom oxygen (orange), the surface oxygen (pink), and the subsurface oxygen (black) spectral regions along the *E*_kin_^PES^ direction. The noncoincident Auger electron spectrum (AES) (gray circles) gives the spectral envelope of all three oxygen species tailing toward the Fermi level.

To relate the experimental O 1s-KVV Auger photoelectron coincidence spectroscopy (APECS) maps of “29” Cu_*x*_O/Cu­(111) surface oxide to our first-principles computational 6 O adatom and 5 O adatom models, we use the oxygen O 2p Projected Local Density of States (PLDOS) in self-convolution to describe for each oxygen species the itinerant band-like two-hole final states. For the O 1s photoelectron coincidence spectroscopy (PECS) data, we employ the (Z + 1) impurity energies for each of the three oxygen species. This allows us to interpret the contributions of the three oxygen species within the O 1s-KVV APECS data in detail. In Figure 5, the left-hand side panels show (a) the experimental O 1s photoelectron coincidence spectroscopy (PECS) data, (c) a direct comparison to fits based on the computed (Z + 1) impurity energies in the 6 O adatom model, and (e) a direct comparison to fits based on the computed (Z + 1) impurity energies in the 5 O adatom model. Adatom oxygen is shown in orange, surface oxygen in pink, and subsurface oxygen in black. For the 6 O adatom model, the computationally derived O 1s core level binding energy shifts are:- surface oxygen 0.954 ± 0.081 eV, subsurface oxygen +1.597 ± 0.249 eV, and adatom oxygen −0.111 ± 0.095 eV. For the 5 O adatom model, the computationally derived O 1s core level binding energy shifts are:- surface oxygen 0.769 ± 0.171 eV, subsurface oxygen +1.323 ± 0.236 eV, and adatom oxygen −0.241 ± 0.165 eV. Panels 5 (c) and (e) show the O 1s PECS fits based on these parameters using coupled asymmetric Voigt line shapes for the oxygen adatom (orange), surface (pink), subsurface (black), and a Shirley background (details in the Supporting Information). The fits based on the 6 and 5 O adatom models are equally good, and with regard to the oxygen chemical speciation, no propensity to either model can be concluded.

**Figure 5:**
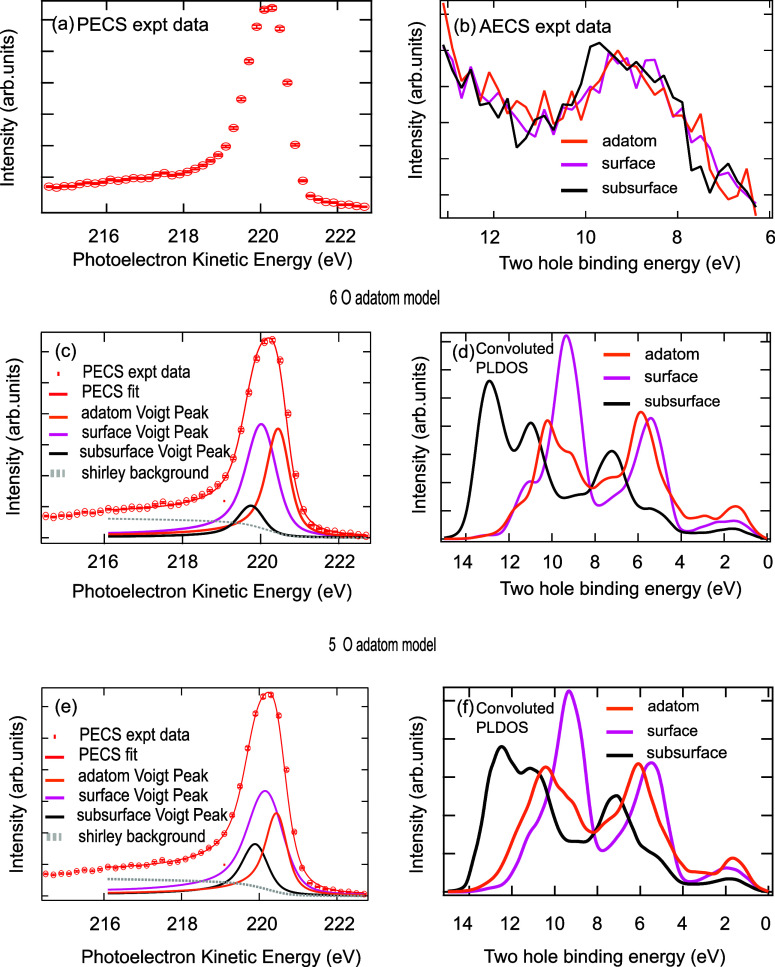
Oxygen speciation based on experimental O 1s-KVV photoelectron coincidence spectra (PECS) and band-like Auger electron coincidence spectra (AECS) in direct comparison to first-principles 6 and 5 O adatom models (Figure 3) (a)) of the “29” Cu_*x*_O/Cu­(111) surface oxide: (a) experimental O 1s PECS data and (b) experimental O 1s AECS spectra for adatom oxygen (orange), surface oxygen (pink), and subsurface oxygen (black). This data is a zoom of Figure 4 (b)) showing particularly the itinerant band-like two hole final states. (c) O 1s PECS fits based on the computed (Z + 1) impurity energies in the 6 O adatom model, (d) shows band-like two-hole final states of each oxygen species from the self-convolution of the O 2p PLDOS computed within the 6 O adatom model, (e) shows O 1s PECS fits, based on the computed (Z + 1) impurity energies in the 5 O adatom model, and (f) shows band-like two-hole final states of each oxygen species from the self-convolution of the O 2p PLDOS computed within the 5 adatom model.

The right-hand side panels of Figure 5 show the itinerant band-like two-hole final states spectral region: In panel Figure 5 (b), we show the experimental AECS band-like adatom oxygen (orange), the surface oxygen (pink), and the subsurface oxygen (black) data as a zoom of Figure 4b). Panels Figure 5(d,f) show band-like two-hole final states of each oxygen species from the self-convolution of the O 2p PLDOS computed within the 6 O adatom (Figure 5d) and 5 O adatom (Figure 5f) models, respectively (details in the Supporting Information and see refs 7 and^32^ ). This self-convoluted PLDOS reflects on the two-hole binding energies of the O 1s-KVV final state containing the itinerant band-like features, however neglecting transition matrix elements and vibrational Franck–Condon factors. We observe for the experimental data and the self-convoluted PLDOS data within the 6 and 5 O adatom model a variation of spectral weight within the band-like two-hole final states spectral distribution: The subsurface oxygen (black) tends toward higher two-hole binding energies. The surface oxygen (pink) and adatom oxygen (orange) are overlapping and at lower two-hole binding energies. These observations allow no preference toward the 6 O adatom or 5 O adatom models. Based on our O 1s-KVV APECS investigation, the oxygen binding energies and electronic structure features associated with these oxygen environments overlap significantly, indicating a gradual and continuous distribution of chemical entities throughout the oxygen electronic structure of the “29” Cu_*x*_O/Cu­(111) surface oxide. However, our study reassures that the experimental O 1s-KVV APECS findings are consistent with the distribution of adatom oxygen (orange), surface oxygen (pink), and subsurface oxygen (black) species, for both the 6 and 5 O adatom models. This could be attributed to the chemical environment of the ‘29” Cu_*x*_O/Cu­(111) surface oxide as removal of the O adatom with a similar occupation site would not change the local electronic structure drastically.

## 3 Conclusion

The formal oxidation state of partially oxidized Cu in the “29” Cu_*x*_O/Cu­(111) surface oxide has been controversial and experimentally difficult to differentiate. We used the uniquely high surface and chemical state selective capabilities of Auger photoelectron coincidence spectroscopy on the copper and oxygen atoms. The spectral observations are computed with first-principles theory for the 6 O adatom and 5 O adatom models that have been proposed for the “29” Cu_*x*_O/Cu­(111) surface oxide. We determine a formal Cu(0.3) oxidation state for the partially oxidized “29” Cu_*x*_O/Cu­(111) surface close to the Cu(0) oxidation state of metallic Cu. This quantitative information about the “29” Cu_*x*_O/Cu­(111) surface oxide has been previously inaccessible by photoelectron spectroscopy or Auger electron spectroscopy alone. For the oxygen sites within the “29” Cu_*x*_O/Cu­(111) surface, we discern adatom, surface, and subsurface oxygen species experimentally and in comparison to first-principles modeling. In this, no propensity toward a 6 or 5 O adatom model seems preferential with regard to the oxygen chemical speciation. Our quantification of the Cu-oxidation state as Cu(0.3) and the distribution of oxygen chemical states in the partially oxidized “29” Cu_*x*_O/Cu­(111) surface oxide clarifies the nature of chemical species that play a governing role in catalysis, corrosion, and electrochemistry of Cu surfaces.

## 4 Experimental Section

### 4.1 Sample preparation for Cu 3p-MVV edge and Cu 2p_3/2_-L_3_VV edge coincidence measurements of Cu, “29” Cu_*x*_O/Cu­(111) surface oxide, Cu_2_O, and CuO

#### 4.1.1 Cu­(111)

A Cu(111) (99,99%) single crystal from MaTecK GmbH was cleaned by repeated cycles of Ar^+^ sputtering and annealing at 900 K until a sharp LEED pattern of the Cu(111) single crystal was obtained. The purity of the crystal was ensured with XPS, which showed only Cu features.

#### 4.1.2 “29” Cu_*x*_O/Cu­(111) Surface Oxide

On the completely clean Cu(111) surface, we dose O_2_ at (5 × 10^–6^ mbar), for 20 min at a temperature of 555 K ± 20 K,^13^ leading to the formation of “29” Cu_*x*_O/Cu­(111) surface oxide (see schematic in Figure 1 of the Supporting Information). LEED measurements were conducted at an energy of 30 eV, substantiating the formation of “29” Cu_*x*_O/Cu­(111) surface oxide.

#### 4.1.3 CuO and Cu_2_O

First, a Cu(110) single crystal by MaTecK GmbH was cleaned by cycles of Ar^+^ sputtering and annealing at 900 K for 5 min until a sharp LEED pattern was observed, and no other elements than copper were detected with XPS. Then, clean Cu(110) was heated on a heating plate in atmosphere at 300°C for 20 min, which is expected to create a thick CuO layer on the surface. On this sample surface, we observed some Carbon with XPS, which was removed by mild Ar^+^ sputtering. After sputtering, the intensity of the characteristic 3d^9^ satellite in Cu 2p_3/2_ XPS decreased, which we attribute to creation of oxygen vacancies and partial reduction of the surface layer due to preferential oxygen sputtering. The surface stoichiometry was then reestablished by dosing oxygen at 2 mbar in situ for 10 min above 300°C. This treatment fully re-established the 3d^9^ satellite, confirming Cu­(II)­O.

Cu_2_O was prepared starting from the CuO thin film on Cu(110) by a combination of Ar^+^ sputtering and heating in situ above 400°C in-vacuum and in 1 × 10^–4^mbar oxygen atmosphere. After all preparation cycles, the Cu­(I) state was confirmed with XPS by comparing the Cu 2p_3/2_ binding energy to tabulated values and by confirming the complete absence of the 3d^9^ satellite which rules out Cu­(II) states. All APECS measurements were performed on well-prepared surfaces, which were characterized by survey XPS to ensure cleanliness and by Cu 2p XPS to confirm the copper oxidation state (see the Supporting Information and ref^4^).

### 4.2 Auger photoelectron coincidence measurements at the storage ring of BESSY II

The Auger Photoelectron coincidence measurements (APECS) were conducted at the coincidence electron spectroscopy for chemical analysis (CoESCA) endstation^24^ at the UE52-PGM beamline of the BESSY II electron storage ring. The base pressure was about 10^–10^ mbar. The two-angle resolved time-of-flight ARToF electron analyzers with their detection efficiency and in combination with the Pulse Picking by Resonant Excitation method at BESSY II provide a high degree of true over accidental coincidences in all the APECS measurements. In detail, the coincidence raw data always contains above the single event limit both “true” as well as “accidental” coincidence events. The true coincidences are pairs of photoelectrons and Auger electrons originating from a single atomic ionization event. Accidental coincidences are electrons arriving at the detectors simultaneously but from different ionization events. We can clock the ARTOF used for photoelectron detection and ARTOF used for Auger electron detection either to the same synchrotron light pulse or to the consecutive ones. For consecutive synchrotron pulses, solely accidental coincidences are obtained, whereas for triggering on the same synchrotron light pulse, a sum of true and accidental coincidences is acquired. Thus, the true coincidences can be isolated by a subtraction routine. For Cu(111), “29” Cu_*x*_O/Cu­(111) surface oxide and Cu_2_O, the coincidences measurements were conducted at 2p_3/2_-L_3_VV edge and also 3p-MVV edge. The accidental, true, and *v*_a_/*v*_t_ ratios for these are given in the Supporting Information. The counts from “29” Cu_*x*_O/Cu­(111) O 1s-KVV measurements and 2p_3/2_-L_3_VV measurements of CuO are also provided in the Supporting Information.

### 4.3 First-principles calculations for Cu and Oxygen

The atomic model of the oxidized “29” Cu_*x*_O/Cu­(111) surface was constructed by first creating a Cu slab having the orientation (111) and a $\sqrt{13}{R46.1}^{{}^{\circ}}\times {7R21.8}^{{}^{\circ}}$ surface unit cell that is 29 times bigger than the Cu(111) surface unit cell. The slab was comprised of 6 layers, each containing 29 Cu atoms. On top of the prepared Cu slab, a (111) slice of the cubic Copper­(I) oxide structure, containing 18 Cu and 18 O atoms for the 6 O adatom model, was placed epitaxially and allowed to relax. For the 5 O adatom model, 18 Cu and 17 O atoms were used. The atomic coordinates for the 3 bottom Cu layers, as well as the slab dimensions (*a* = 9.255 Å, *b* = 17.968 Å, and *c* = 23.498 Å) and shape (α = β = 90°, γ = 84.3°), were kept fixed during the relaxation procedure. The electronic structure and total energy calculations were based on density functional theory and were performed in the generalized gradient approximation^37^ using the Vienna Ab Initio Simulation Package (VASP),^20^ projector augmented wave (PAW) type pseudopotentials,^3,21^ and a plane-wave basis set with a kinetic energy cutoff *E*_c_ = 500 eV. Structure stability was also tested against atomic degrees of freedom (such as O- or Cu-termination of the oxidized surface as well as the presence of Cu vacancies in the substrate layer) using chemical potentials of O and Cu species computed for the Cu–Cu_2_O equilibrium. These tests revealed that the original structure was the lowest in energy. The optimum atomic structure was kept fixed when core excitations in the near-surface Cu atoms were simulated. In these simulations, each of the 76 Cu atoms (18 + 29 + 29) in the three topmost Cu layers was subsequently transmuted to an atomic species *Z* + 2 (Ga), thus mimicking the two-hole final state.^14,27^ The surface core level shifts (SCLS) were computed by comparing the total energies of the slabs containing a *Z* + 2 impurity in the surface or subsurface layers with the total energy of the slab containing the impurity in the bulk-like (fourth from bottom) layer. The supercell remained neutral throughout the simulations. Nevertheless, possible effects of spurious dipole interactions between the periodically repeating slabs were checked and found to be negligible. During the structural optimization, a sparse Monkhorst–Pack^30^ mesh of *k*-points, 5 × 3 × 2, was used for the Brillouin zone integration, while in the density of states (DOS) and in the final calculations of total energy, the mesh density was increased to 9 × 7 × 3. The site-projected DOS was computed for all 18 oxygen atoms in the structure for the 6 O adatom model and for all 17 oxygen atoms in the structure for the 5 O adatom model. Three groups of O atoms on the oxidized Cu(111) surface have been analyzed for both the 6 and 5 O adatom model. In the 6 and 5 O adatom model, the topmost O (group 1) are 3-fold coordinated to the underlying Cu atoms while facing vacuum on the upper side. The adatom O (group 3) atoms in the 6 O adatom model are also similarly 3-fold coordinated, whereas the O adatoms in the 5 O adatom model are predominantly 3-fold coordinated (except one O-adatom that has migrated too close to a hexagonal ring). Thus, the DFT-calculated O-2p projected local densities of states of topmost O (group 1) and adatom O (group 3) appear very similar to each other for both the 6 and 5 O adatom model. The coordination of subsurface O (group 2) atoms is 4-fold for both 6 and 5 O adatom model: they are coordinated to three Cu atoms on the upper side and to one Cu atom beneath. This difference is reflected in their electronic structure: O-2p PLDOS peaks in the energy range from −8 to −3 eV relative to the Fermi level are differently shaped and shifted somewhat down in energy (see the Supporting Information). This shows that local coordination has the main effect on the electronic structure of O atoms: O-2p PLDOS allows one to clearly distinguish between 3-fold-coordinated and 4-fold-coordinated O atoms. For determination of (Z + 1) SCLS, each of the 18 O atoms in the 6 O adatom model and 17 O atoms in the 5 adatom model was subsequently considered as an impurity with a 1s core hole, in VASP. In the model, the impurity with a core hole was replaced with an equivalent (Z + 1) impurity, that is (Fluorine), F. The surface core level shifts were calculated from the total energy and expressed relative to the average level of the adatom O. The mean surface core level shift and the standard deviation for the average within each group of O atoms for the 6 O adatom model are as such: For surface oxygen, the values are 0.954 ± 0.081 eV, subsurface oxygen +1.597 ± 0.249 eV, and adatom oxygen −0.111 ± 0.095 eV. The mean surface core level shift and the standard deviation for the average within each group of O atoms for the 5 O adatom model are as such: surface oxygen 0.769 ± 0.171 eV, subsurface oxygen +1.323 ± 0.236 eV, and adatom oxygen −0.241 ± 0.165 eV.

## Supplementary Material



## Notes

Additional XPS and LEED measurements, fitting methods, and experimental details including experimental settings (PDF)
