# Supplementary material for: Character of Cu and O in partially oxidized “29” Cu x O/Cu(111) from Auger Photoelectron Coincidence Spectroscopy
Source: J Am Chem Soc. 2026 Jul 31;148(31):33548–58. doi: 10.1021/jacs.6c08886 (PMC13474937; doi:10.1021/jacs.6c08886)
Supplement: Supplementary file 1 [file ja6c08886_si_001.pdf]

Character of Cu and O in partially oxidized 29 $Cu_xO/Cu(111)$ from Auger Photoelectron Coincidence Spectroscopy

Swarnshikha Sinha[1,2,3], Danilo Kühn[2,3], Fredrik O. L. Johansson[3,4], Andreas Lindblad[3,4], Pavel A. Korzhavyi[5], Nils Mårtensson[3,4], and Alexander Föhlisch*[1,2,3]

[1]Helmholtz Zentrum Berlin für Materialien and Energie GmbH, Albert-Einstein Straße 15, 12489 Berlin, Germany
[2]Universtät Potsdam, Institut für Physik und Astronomie, Karl-Liebknecht- Straße, 14476, Potsdam, Germany
[3]Uppsala-Berlin Joint Laboratory on Next Generation Photoelectron Spectroscopy, 12489 Berlin, Germany
[4]Division of X-ray Photon Science, Department of Physics and Astronomy, Uppsala University, P.O. Box 256, 751 20 Uppsala, Sweden
[5]Department of Materials Science and Engineering, KTH Royal Institute of Technology, Brinellvägen 23, 100 44 Stockholm, Sweden

1 Supporting Information

On the freshly prepared “29" $Cu_xO/Cu(111)$ surface Oxide (schematic of preparation in Fig 1), we conducted Low Energy Electron Diffraction (LEED) study at an energy of 30 eV, see Fig 1. LEED characterization confirmed the construction of “29" $Cu_xO/Cu(111)$ surface oxide.

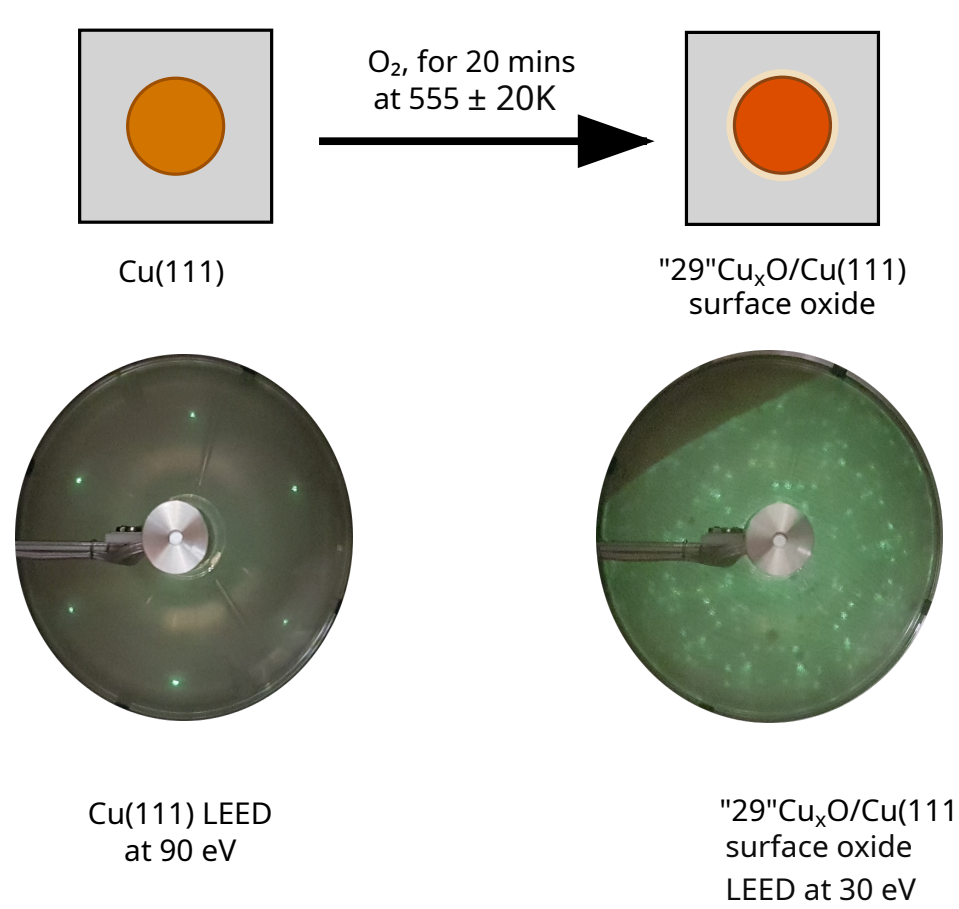

Figure 1: Preparation schematic of the “29" $Cu_xO/Cu(111)$ surface oxide and LEED characterization of Cu(111) and “29" $Cu_xO/Cu(111)$ surface oxide recorded at 90 eV and 30 eV primary electron energies respectively.

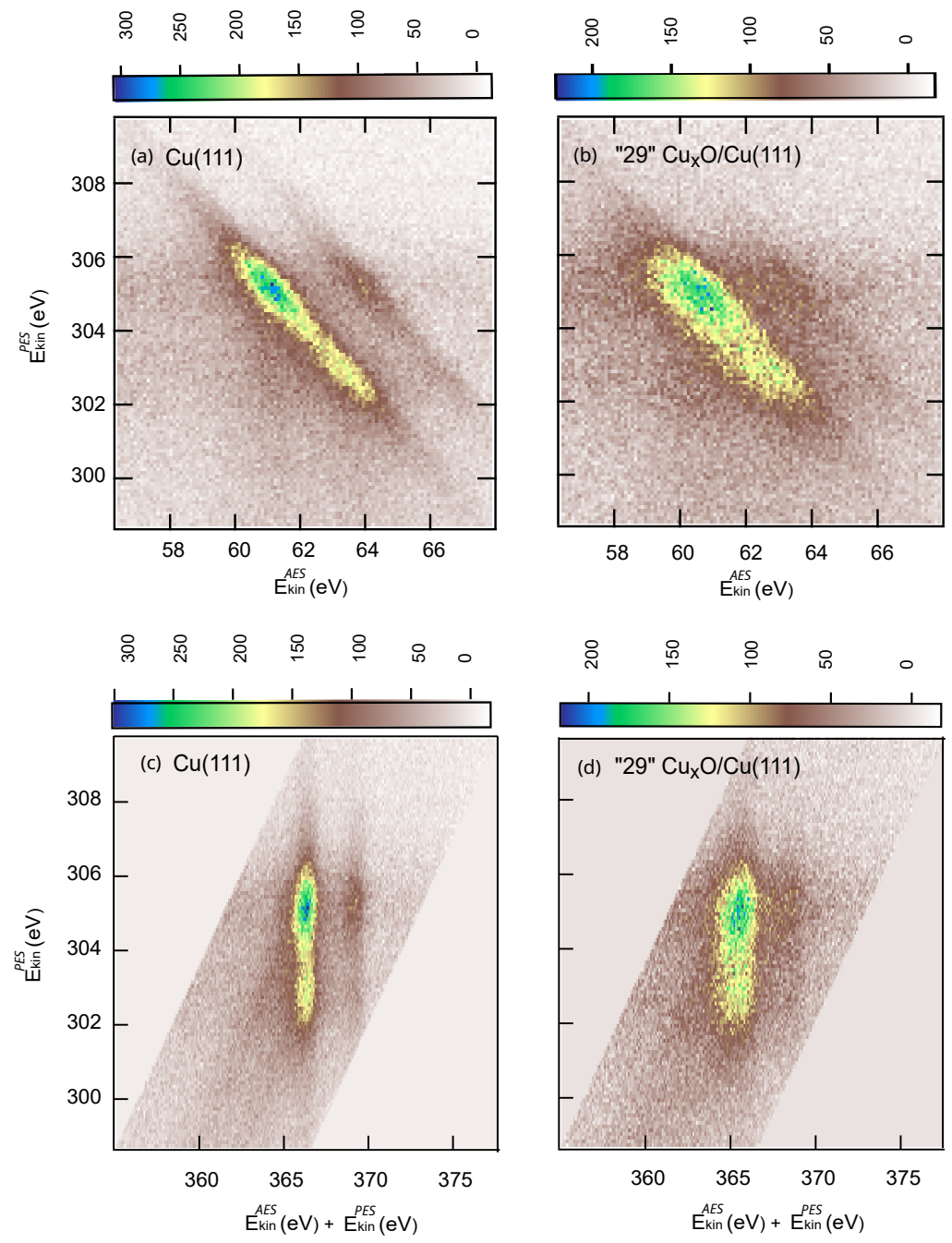

Figure 2: The panels (a) and (b) are the coincidence measurements of the Cu(111) and "29" Cu_xO/Cu(111) surface oxide at the Cu 3p-MVV edge in E_{kin}^{AES} and E_{kin}^{PES} energy scale. Panels(c) and (d) are projection of panels (a) and (b) in $E_{kin}^{AES} + E_{kin}^{PES}$ energy scale

1.1 Coincidence measurements of "29" Cu_xO/Cu(111) surface oxide and Cu(111) and transformation to $E_{kin}^{AES} + E_{kin}^{PES}$ energy scale

The APECS maps in Fig 2 show intensities of coincidence Auger/photoelectron pairs plotted against E_{kin}^{AES} on the x-axis and E_{kin}^{PES} on the y-axis for the Cu(111) and "29" Cu_xO/Cu(111) surface oxide. There are diagonal features within the 2D coincidence maps. They reflect the localized atomic nature of the Cu 3p-MVV process as the emission of the photoelectron and the Auger electron remains a coherent process preserving the energy sum. After transforming the x-axis to energy sum, one can directly obtain the two-hole states which follow the equation ($E_{kin}^{AES} + E_{kin}^{PES} = h\nu - E_{2H}$), where E_{2H} is the two-hole final state binding energy. E_{kin}^{AES} and E_{kin}^{PES} are calibrated to the Fermi level of the pure copper crystal [5].

1.2 XPS and AES measurements of “29" $Cu_xO/Cu(111)$ surface oxide and Cu(111)

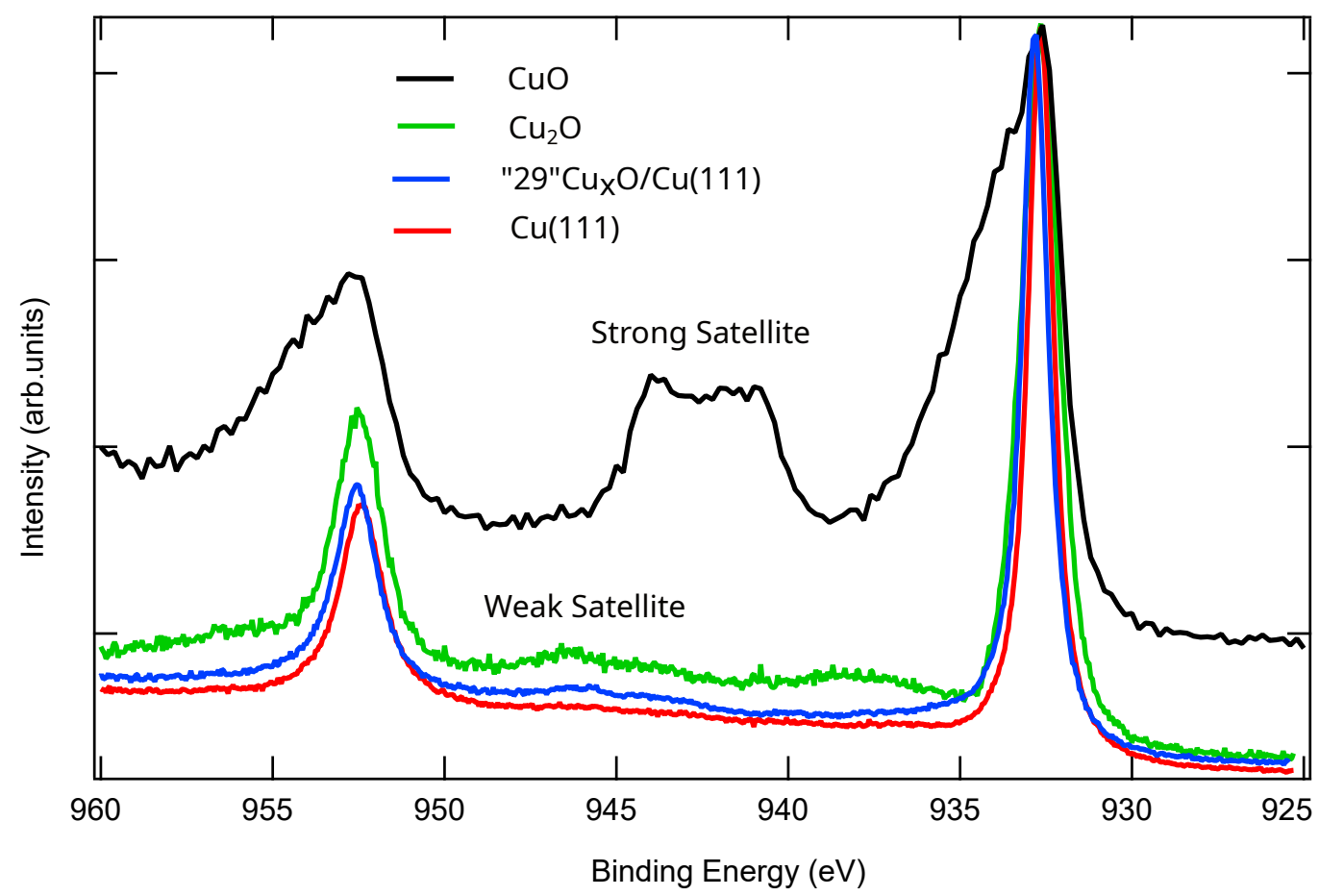

Figure 3: Cu 2p photoemission spectra of cupric oxide (CuO), cuprous oxide Cu_2O, “29" $Cu_xO/Cu(111)$ surface oxide and Cu(111)

Fig. 3 presents the Cu 2p photoemission spectra of the as prepared cupric oxide (CuO), cuprous oxide Cu_2O, “29" $Cu_xO/Cu(111)$ surface oxide and Cu(111). The XPS spectra reveal characteristic features of the copper oxide phases: CuO exhibits strong $3d^9$ shake-up satellites, Cu_2O displays weak satellite intensity, and the "29" $Cu_xO/Cu(111)$ surface oxide shows intermediate behavior between Cu_2O and metallic copper. The measured Cu $2p_{3/2}$ binding energies agree with literature [1]. This validates the phase purity of cupric oxide (CuO), cuprous oxide (Cu_2O), and the "29" $Cu_xO/Cu(111)$ surface oxide structure.

1.3 Comparison of coincidence measurement of Cu $2p_{3/2}$, Cu $3p_{3/2}$ and non coincident Auger LVV of the “29" $Cu_xO/Cu(111)$ surface oxide.

We performed coincidence measurements on Cu $2p_{3/2}$- L_3VV and Cu $3p_{3/2}$ -M_3VV of “29" $Cu_xO/Cu(111)$ surface oxide and put it in comparison to the non-coincident conventional Auger LVV spectra of “29" $Cu_xO/Cu(111)$ surface oxide in Fig. 4. The Auger LVV has been coincided on top of the Cu $2p_{3/2}$, Cu $3p_{3/2}$AECS to match the peak maximum. The non-coincidence LVV Auger spectrum shows structures at about 20 eV and -2 eV two-hole binding energies which are related to Cu $2p_{1/2}$ core ionization. Hence, these structures are absent in the coincidence spectra. The main feature at about 15 eV, which is the ^1G final state of the Cu $3d^8$ atomic multiplet, has a similar width in the non-coincidence AES and in the $2p_{3/2}/L_3VV$ AECS spectra. The same feature appears broadened towards higher two-hole binding energy in the very surface sensitive $3p_{3/2}/M_3VV$ AECS spectrum, indicating the presence of more than one Cu entities. We attribute this to a relatively increased signal from copper in the top layer of the“29" $Cu_xO/Cu(111)$ surface oxide, which has a chemical shift to higher binding energy, due to the smaller probing depth of the 3p APECS measurement compared to 2p APECS (see discussion of the LVV vs MVV probing depth for pure copper in [5]).

1.4 Calculation of Depth-Dependent Intensity Contributions

We calculated depth-dependent intensity contributions for our three layer fit model of the two-hole spectra. The depth profile of the signal arising from first, second and bulk layer of the “29" $Cu_xO/Cu(111)$ surface oxide is obtained by integrating the signal over the interval [p,q] using an exponential attenuation model. The intensity emitted from a layer of material between depths p and q is proportional to the integral as given by the equation:

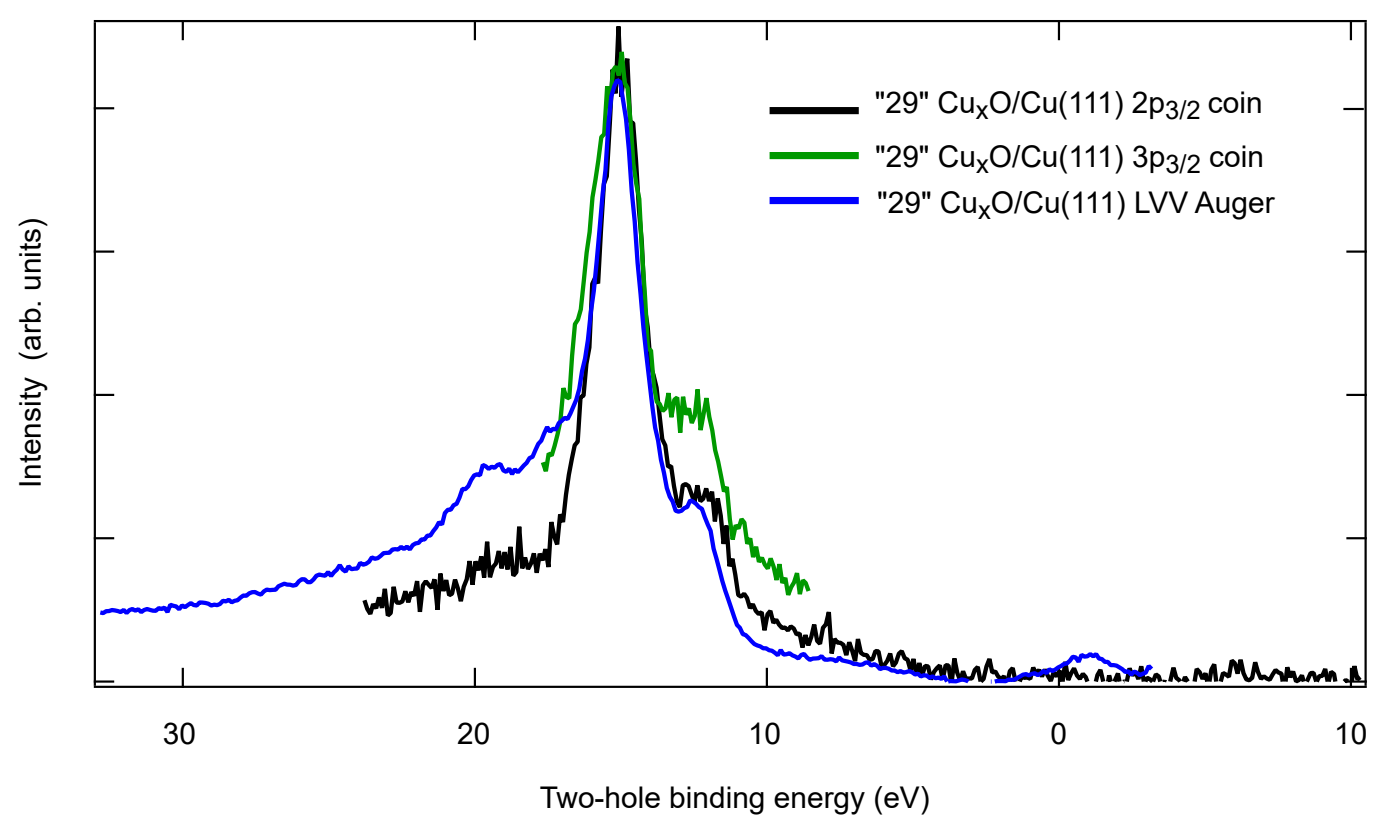

Figure 4: Comparison of Cu $2p_{3/2}$, Cu $3p_{3/2}$ AECS and Auger LVV of the "29" $Cu_xO/Cu(111)$ surface oxide. The Auger LVV has been coincided on top of the Cu $2p_{3/2}$, Cu $3p_{3/2}$AECS to match the peak maximum.

$$I = I_0 \int_p^q e^{-x/\Delta}\, dx$$

Here, Δ is the mean escape depth of the material, obtained from $\Delta = \lambda cos\theta$, where the inelastic mean free path λ is obtained from [4] and is 0.497 nm for Auger electron kinetic energy (60 eV) and 0.785 nm for photoelectron kinetic energy (300 eV), applied under a Auger electron detection cone with a central angle of 49° and a photoelectron detection cone with a central angle of 59°.

Table 1: p and q depths of Layer 1, Layer 2 and Layer 3 + bulk (in nm) for the "29" $Cu_xO/Cu(111)$ surface oxide obtained by atomic density and depth profiling of each layer

	p	q
Cu 1	0	0.207
Cu 2	0.207	0.414
Cu 3 + Bulk	0.414	∞

To obtain the layer thickness (interlayer spacing) we use a lattice constant value of 0.359 nm for the three layers of "29" $Cu_xO/Cu(111)$ surface oxide constructed over Cu(111) Surface and the equation d_{hkl} =a/$\sqrt{h^2 + k^2 + l^2}$ where h,k,l are the miller indices of Cu(111) surfaces and a is the lattice constant value of Cu. The integration limits (p,q), obtained are the layer thicknesses (interlayer spacing), in nm for the three layers of Cu surfaces and are tabulated in Tab.1. Further, the atomic densities (No of atoms/unit Area) of each layer were calculated from the lattice parameters derived from VASP. The depth-dependent intensities (in %) from the first layer, second layer, and the bulk (including the third layer) were determined by multiplying the respective intensities from the exponential attenuation model with their atomic densities. There are 18 Cu atoms on the first layer and 29 Cu atoms on the second, third and bulk layers. The intensity values was found to be 57.3% for Cu 1, 29.2% for Cu 2 and 13.5% Cu 3 + bulk. The relative intensity for (Cu 1/Cu 3 +bulk) was found to be 4.24 and the relative intensity for (Cu 2/Cu 3 + Bulk) was found to be 2.16. These relative intensities were used in the depth dependent intensity model for the Cu 3p $_{3/2}$-M_3VV AECS spectra in both 6 and 5 O adatom system.

1.5 True and Accidental Counts of the APECS measurement

Table 2: True and accidental counts of all APECS measurements are given with v_a/v_t ratio

Cu $2p_{3/2}$-L_3VV	Accidental Counts	True Counts	v_a/v_t Ratio	Cu 3p-MVV & O1s-KVV	Accidental Counts	True Counts	v_a/v_t Ratio
Cu 111	54298	62196	0.87	Cu111	654800	520910	1.26
"29" Cu_xO/Cu(111)	712305	234380	3.04	Cu 3p 29	986640	461594	2.14
Cu_2O	175443	134005	1.31	Cu 3p	403015.5	100776	4.00
CuO	3885072	941988	4.12	O1s	438056	344963	1.27

1.6 Asymmetric Voigt lineshapes fit to the PECS of "29" Cu_xO/Cu(111) surface oxide at the O1s-KVV edge and the self convoluted PLDOS of the three oxygen species of "29" Cu_xO/Cu(111) surface oxide for both the 6 and 5 O adatom model

Table 3: Fit values for the PECS experimental spectra modeled with three Voigt line shapes and asymmetry for the 6 O adatom model in Fig. 5(c) of the main text. The lorentzian and SCLS values are constrained. Asymmetry value was found to be 0.09±0.01.

Fit Parameters	Peak adatom	Peak surface	Peak subsurface
Gaussian Width FWHM(eV)	0.43	0.66	0.52
Lorentzian Width FWHM(eV)	0.18	0.18	0.18
Energy Position (eV)	220.48	220.06	219.78
Intensity	5200	6600	1658

Table 4: Fit values of the PECS experimental spectra modeled with three Voigt line shapes and asymmetry for the 5 O adatom model in Fig. 5(e) of the main text. The lorentzian and SCLS values are constrained. Asymmetry value was found to be 0.13 ±0.01.

Fit Parameters	Peak adatom	Peak surface	Peak subsurface
Gaussian Width FWHM(eV)	0.38	0.81	0.52
Lorentzian Width FWHM(eV)	0.18	0.18	0.18
Energy Position (eV)	220.48	220.22	219.94
Intensity	3742	6766	2663

In our investigation of the "29" Cu_xO/Cu(111) surface oxide, we performed a comprehensive spectroscopic analysis utilizing O1s KVV coincidence measurements. The procedure involved integration along the Auger electron kinetic energy E_{kin}^{AES} axis of the 2D coincidence map (see Fig 4(a) main text) to extract the Photoelectron Coincidence Spectra (PECS) in the Fig.5 (a) of the main text. Our (Z+1) Surface Core Level Shift (SCLS) calculations, and Projected Local Density of states (PLDOS) analysis, revealed the presence of three distinct oxygen species within the structure. We subsequently fitted the experimental PECS of Fig.5 (a) using a model comprising three Voigt profiles with asymmetry corresponding to these oxygen species, see Fig.5 (c) and (e) of main text. All fitting parameters were optimized independently, while incorporating energy shifts derived from the (Z+1) SCLS calculations and Lorentzian width as initial constraints. The

(Z+1) SCLS herein were scaled by a factor of 0.5 to fit in the overall distribution. The fit values are presented in Tab.3 and Tab.4. The Lorentzian width FWHM was set to literature value of 0.18 eV [3, 2] for both the 6 and 5 O adatom model. The asymmetry values for the 6 and 5 O adatom model was found to be 0.09 ±0.01 and 0.13 ±0.01. The Gaussian width (FWHM) in the 6 O adatom model was found to be 0.43 ±0.02 eV for the adatom, 0.66 ±0.1 eV for surface, 0.52 ±0.1 eV for the subsurface oxygen. The Gaussian width (FWHM) in the 5 O adatom model was found to be 0.38 ±0.1 eV for the adatom, 0.81 ±0.1 eV for surface, 0.52 ±0.1 eV for the subsurface oxygen. Further, self-convoluted PLDOS were obtained (see Fig 5 (d) and (f) of the main text) from O 2p PLDOS presented in Fig. 5 (a) and (b) for both the 6 and 5 O adatom model.

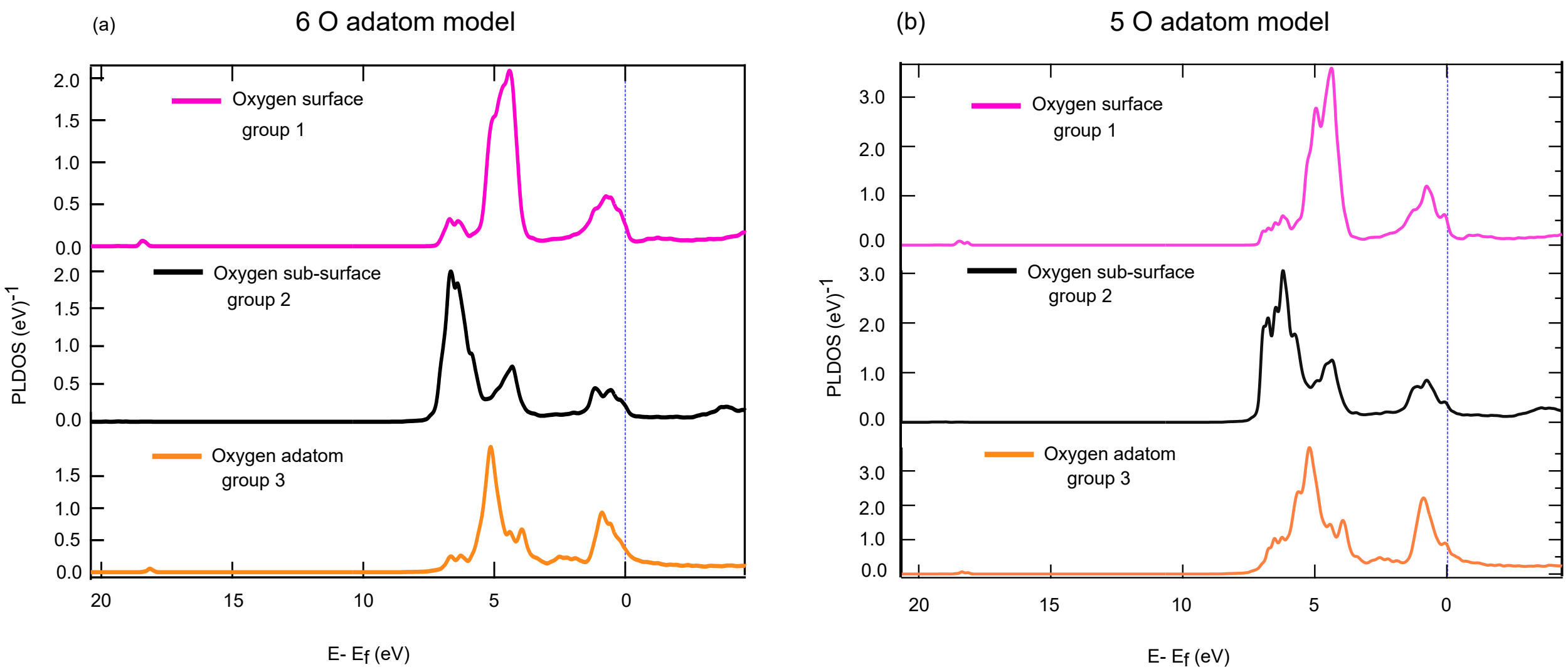

Figure 5: Panels (a) and (b) give the O 2p PLDOS for each oxygen species calculated with Density functional theory for the 6 and 5 O adatom model.

1.7 Experimental Settings

The experimental settings of the Cu $2p_{3/2}$-L_3VV measurements of Cu(111), “29" Cu_xO/Cu(111), Cu_2O and CuO are given in Tab. 5 and Cu $3p_{3/2}$-M_3VV measurements of Cu(111), Cu_xO/Cu(111) and Cu_2O, as well as O1s KVV of “29" Cu_xO/Cu(111) are given in Tab. 6. At some point one of the spectrometers (”ARTOF-1”) was replaced by a new spectrometer (”ARTOF-3”). ”ARTOF-2” and ”ARTOF-3” are the same spectrometer model.

Table 5: The photon energy used for all the measurements for Cu $2p_{3/2}$-L_3VV are presented. All measurements were carried out with horizontal polarisation

.

Surfaces	Cu $2p_{3/2}$-L_3VV		
	Energy (eV)	Detector Settings<br>(Central Axis Angle<br>with respect to the sample normal)<br>&<br>Edge Measured<br>&<br>Energy Window, Full cone acceptance angle	Detector Settings<br>(Central Axis Angle<br>with respect to the sample normal)<br>&<br>Edge Measured<br>&<br>Energy Window, Full cone acceptance angle
Cu 111	1100	ARTOF-3 (44°)<br><br>CuL_3VV<br><br>4%, 56°	ARTOF-2 (64°)<br><br>Cu$2p_{3/2}$<br><br>4%, 56°
"29" Cu_xO/Cu(111)	1100	ARTOF-3 (44°)<br><br>CuL_3VV<br><br>4%, 56°	ARTOF-2 (64°)<br><br>Cu$2p_{3/2}$<br><br>4%, 56°
Cu_2O	1250	ARTOF-3 (44°)<br><br>CuL_3VV<br><br>4%, 56°	ARTOF-2 (64°)<br><br>Cu$2p_{3/2}$<br><br>7%, 50°
CuO	1250	ARTOF-3 (44°)<br><br>CuL_3VV<br><br>4%, 56°	ARTOF-2 (64°)<br><br>Cu$2p_{3/2}$<br><br>7%, 50°

Table 6: The photon energy used for all the measurements for Cu 3p MVV & O1s-KVV are presented. All measurements ere carried out with horizontal polarisation
.

Surfaces	Cu 3p MVV & O1s-KVV		
	Energy (eV)	Detector Settings<br>(Central Axis Angle<br>with respect to the sample normal)<br>&<br>Edge Measured<br>&<br>Energy Window, Full cone acceptance angle	Detector Settings<br>(Central Axis Angle<br>with respect to the sample normal)<br>&<br>Edge Measured<br>&<br>Energy Window, Full cone acceptance angle
Cu 111	380	ARTOF-1 (49°)<br><br>Cu MVV<br><br>10%, 30°	ARTOF-2 (54°)<br><br>Cu 3p<br><br>4%, 56°
"29" Cu_xO/Cu(111)	380	ARTOF-1 (49°)<br><br>Cu MVV<br><br>20%, 30°	ARTOF-2 (54°)<br><br>Cu3p<br><br>4%, 56°
Cu_2O	380	ARTOF-1 (49°)<br><br>Cu MVV<br><br>20%, 30°	ARTOF-2 (54°)<br><br>Cu3p<br><br>4%, 56°
"29" Cu_xO/Cu(111)	750	ARTOF-3 (44°)<br><br>O KVV<br><br>4%, 56°	ARTOF-2 (64°)<br><br>O1s<br><br>4%, 56°

References

[1] Mark H Engelhard, Donald R Baer, Alberto Herrera-Gomez, and Peter Sherwood. "Introductory guide to backgrounds in XPS spectra and their impact on determining peak intensities". In: *Journal of Vacuum Science & Technology A* 38.6 (2020).

[2] A Föhlisch, Nial Wassdahl, J Hasselström, O Karis, Dietrich Menzel, N Mårtensson, and Anders Nilsson. "Beyond the chemical shift: vibrationally resolved core-level photoelectron spectra of adsorbed CO". In: *Physical review letters* 81.8 (1998), p. 1730.

[3] M Neeb, J-E Rubensson, M Biermann, and W Eberhardt. "Coherent excitation of vibrational wave functions observed in core hole decay spectra of O2, N2 and CO". In: *Journal of electron spectroscopy and related phenomena* 67.2 (1994), pp. 261–274.

[4] Hiroshi Shinotsuka, Shigeo Tanuma, Cedric J Powell, and David R Penn. "Calculations of electron inelastic mean free paths. X. Data for 41 elemental solids over the 50 eV to 200 keV range with the relativistic full Penn algorithm". In: *Surface and Interface Analysis* 47.9 (2015), pp. 871–888.

[5] Swarnshikha Sinha, Danilo Kühn, Fredrik OL Johansson, Andreas Lindblad, Nils Mårtensson, Börje Johansson, Pavel A Korzhavyi, and Alexander Föhlisch. "Collective contributions to the atomic Auger photoelectron coincidences on the (100),(110) and (111) facets of copper". In: *Scientific Reports* 15.1 (2025), p. 26411.
